# Effect of an Exercise and Nutrition Program on Quality of Life in Patients With Atrial Fibrillation: The Atrial Fibrillation Lifestyle Project (ALP)

**DOI:** 10.1016/j.cjco.2022.04.004

**Published:** 2022-04-27

**Authors:** Jesse Bittman, Cynthia J. Thomson, Lloyd A. Lyall, Stephanie L. Alexis, Eric T. Lyall, Sebastian L. Cannatella, Mahasti Ebtia, Alexander Fritz, Benjamin K. Freedman, Nooshin Alizadeh-Pasdar, Joan M. LeDrew, Teddi L. Orenstein Lyall

**Affiliations:** a Division of General Internal Medicine, Department of Medicine, University of British Columbia, Vancouver, British Columbia, Canada; b Faculty of Health Sciences, University of the Fraser Valley, Chilliwack, British Columbia, Canada; c School of Humanities and Sciences, Stanford University, Stanford, California, USA; d Faculty of Medicine, University of British Columbia, Vancouver, British Columbia, Canada; e Faculty of Applied Science, University of British Columbia, Vancouver, British Columbia, Canada; f Department of Biochemistry and Microbiology, Faculty of Science, University of Victoria, Victoria, British Columbia, Canada; g Division of Cardiology, Department of Medicine, University of British Columbia Vancouver, British Columbia, Canada; h Department of Science, University of Victoria, Victoria, British Columbia, Canada; i School of Kinesiology, Faculty of Health Sciences, Western University, London, Ontario, Canada; j Faculty of Land and Food Systems, University of British Columbia, Vancouver, British Columbia, Canada; k Cardiac Rehabilitation Services, Richmond Hospital, Vancouver Coastal Health, Richmond, British Columbia, Canada

## Abstract

**Background:**

Studies of separate exercise and weight loss interventions have reported improvements in quality of life (QoL) or reduction in atrial fibrillation (AF) burden. We investigated the impact of a structured exercise, nutrition, and risk-factor-modification program on QoL and AF burden.

**Methods:**

In this trial, 81 successive patients with body mass index > 27 kg/m^2^ and nonpermanent AF were randomized to an intervention (n = 41) or control group (n = 40). The intervention consisted of cardiovascular risk management and a 6-month nutrition and exercise program, followed by a 6-month maintenance program. All participants received usual AF care. The primary end-point was QoL at 6 and 12 months.

**Results:**

At 6 months, we observed improved QoL among patients in the intervention group, relative to that among control-group patients (intervention (I) n = 34, control (C) n = 38) in the 36-item Short Form Survey Instrument scores on the subscales of vitality (I: 13.2 ± 20.4; C: 1.0 ± 14.9, P < 0.001), social functioning (I: 14.7 ± 24.1; C: 2.4 ± 21.2, *P* = 0.018), emotional well-being (I: 5.5 ± 14.1 ; C: –1.0 ± 13.3, *P* = 0.017), and general health perceptions (I: 8.1 ± 12.3; C: 2.7 ± 13.3, *P* = 0.009). At the 6-month follow-up, improvement in the scores on the subscales of vitality (*P* = 0.021) and emotional well-being (*P* = 0.036) remained significant. The burden of AF as measured by Holter monitor and Toronto AF symptom score was not significantly changed.

**Conclusions:**

A structured exercise and nutrition program resulted in significant sustained improvements in QoL, without reduction in AF burden. This type of program may provide an additional treatment for people with impaired QoL due to AF.

## Graphical abstract

**Figure:**
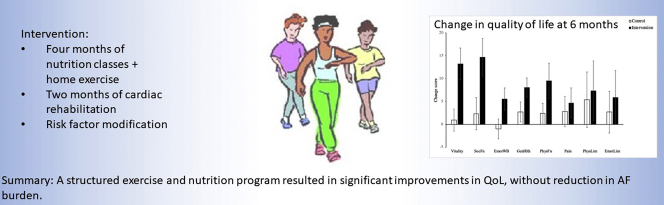

## Résumé

**Introduction:**

Des études sur des interventions distinctes d’exercice et de perte de poids ont montré des améliorations de la qualité de vie (QdV) ou la réduction du fardeau de la fibrillation auriculaire (FA). Nous avons examiné les répercussions d’un programme structuré d’exercice, d’alimentation et de modification des facteurs de risque sur la QdV et le fardeau de la FA.

**Méthodes:**

Dans le présent essai, nous avons réparti de façon aléatoire 81 patients successifs dont l’indice de masse corporelle était > 27 kg/m^2^ et la FA était non permanente à une intervention (n = 41) ou à un groupe témoin (n = 40). L’intervention a consisté en la prise en charge du risque cardiovasculaire et un programme d’alimentation et d’exercice de six mois, et a été suivie d’un programme de maintien de six mois. Tous les participants ont reçu les soins usuels relatifs à la FA. Le principal critère d’évaluation était la QdV après six mois et 12 mois.

**Résultats:**

Après six mois, nous avons observé la QdV chez les patients du groupe d’intervention par rapport à celle des patients du groupe témoin (intervention [I] n = 34, témoin [C] n = 38) selon les scores de la version abrégée du questionnaire de 36 items aux sous-échelles sur la vitalité (I : 13,2 ± 20,4; C : 1,0 ± 14,9, P < 0,001), le fonctionnement social (I : 14,7 ± 24,1; C : 2,4 ± 21,2, *P* = 0,018), le bien-être émotionnel (I : 5,5 ± 14,1 ; C : –1,0 ± 13,3, *P* = 0,017), et les perceptions de la santé générale (I : 8,1 ± 12,3; C : 2,7 ± 13,3, *P* = 0,009). Au suivi après six mois, l’amélioration des scores aux sous-échelles sur la vitalité (*P* = 0,021) et le bien-être émotionnel (*P* = 0,036) demeurait significative. Le fardeau de la FA selon le moniteur Holter et le score selon la *Toronto Atrial Fibrillation Severity Scale* n’avait pas changé de façon significative.

**Conclusions:**

Un programme structuré d’exercice et d’alimentation a donné lieu à des améliorations significatives et soutenues de la QdV, sans réduire le fardeau de la FA. Ce type de programme peut constituer un traitement supplémentaire aux personnes qui connaissent une diminution de leur QdV en raison de la FA.

Atrial fibrillation (AF) is the most common sustained arrhythmia, and it is associated with increased risk of stroke, increased overall mortality, and decreased quality of life (QoL).^1^^,^^2^ Traditionally, guideline recommendations have focused on reducing the risk of stroke and other cardiovascular complications with use of anticoagulation and heart rate control. The most recent Canadian guidelines, however, include recommendations on management of modifiable risk factors, including inactivity and obesity, to reduce arrhythmia, cardiovascular complications, and symptoms.^1^

Approximately 60% of nonvalvular AF cases are associated with modifiable risk factors, such as hypertension, obesity, and diabetes.^3^ Treating these risk factors decreases morbidity and mortality in patients with AF.^3^, ^4^, ^5^ Lifestyle interventions have been found to have a positive impact on AF, but evidence for this comes largely from short-term or nonrandomized trials. Exercise and weight loss, for example, both have been shown to decrease AF burden in short-term randomized trials.^6^, ^7^, ^8^, ^9^ Additionally, a systematic review found that exercise improved QoL in the short term^10^; however, the longer-term impact of exercise on QoL and the impact of weight loss on QoL are not well studied.^11^, ^12^, ^13^

Evidence supporting comprehensive lifestyle intervention and risk-factor management, such as cardiac rehabilitation (CR), is even more limited. The nonrandomized **A**gg**r**essive **R**isk Factor R**e**duction **St**udy for **A**trial **F**ibrillation and Implications for the Outcome of Ablation (Arrest AF), which employed aggressive risk-factor modification, with exercise and weight loss components, resulted in improved arrhythmia-free survival after AF ablation.^5^ Similarly, a retrospective review found fewer AF-related emergency room visits and cardiovascular hospitalizations in CR participants, compared to the number among those cared for by usual specialist-based care.^14^ However, a recent systematic review found a variable impact of CR on QoL and identified a lack of evidence regarding effect of CR on AF burden.^15^

The current study employs a randomized design to assess the effect of a 6-month intensive program of in-center exercise, nutrition, stress management, and medical management, followed by a 6-month home-based maintenance program (ie, intervention group), compared to usual care (ie, control group) on AF burden and QoL in patients with paroxysmal AF.

## Methods

### Study design

The Atrial Fibrillation Lifestyle Project is a parallel-group randomized controlled trial conducted in Richmond, British Columbia, between August 2018 and June 2020. Consecutive patients referred to 4 cardiologists were screened for eligibility. Eligible participants provided informed consent, and the institutional research ethics board approved procedures. The trial was registered with ClinicalTrials.gov (NCT03724383).

### Participant characteristics

Eligible patients were 19 to 75 years of age, had nonpermanent, nonvalvular AF and a body mass index (BMI) ≥ 27 kg/m^2^ or an increased waist circumference based on ethnicity-specific values recommended by the Canadian Diabetes Association,^16^ Participants were required to be in sinus rhythm at the time of randomization. Participants were excluded if they had any of the following: valvular AF (any mechanical heart valve or moderate-to-severe mitral stenosis),^17^ severe aortic valve stenosis, prior mitral valve surgery, severe mitral valve regurgitation, left ventricular ejection fraction < 40%, weight > 136 kg, New York Heart Association class 3-4 heart failure, hypertrophic cardiomyopathy, any absolute contraindications to exercise testing,^18^ or AF ablation within 3 months prior to randomization.

### Participant enrollment

Participants were enrolled in 2 cohorts in series, as follows: cohort 1 (n = 40), randomized August 6, 2018, with intervention starting September 3, 2018; and cohort 2 (n = 41), randomized May 20, 2019, with intervention starting June 17, 2019. Both cohorts had a 2-week gap after 4 months so that cohort 1 would not receive classes during the Christmas holiday period.

All participants received standard AF care, according to Canadian Cardiovascular Society guidelines,^1^ and instruction on Canada’s Food Guide and Physical Activity Guidelines. All participants also received a Fitbit activity tracker (Fitbit Inc., San Francisco, CA), an in-person instructional tutorial, and a wellness tracker to record physical activity.

### Randomization and blinding

After baseline testing, 81 participants were randomized and stratified within their cohort. We employed computer-generated block randomization to create varying block randomization. All data were blinded, except the follow-up medical history. Participants completed all surveys independently.

### Experimental intervention

The intervention consisted of a 6-month active intervention followed by a 6-month home-based maintenance phase.

The active intervention phase (phase 1) consisted of the following: part 1—a 4-month dietician-led nutrition program; part 2—a 4-month home-based exercise program; and part 3—a 2-month supervised cardiac rehab program consisting of twice-weekly moderate-intensity interval training at the Richmond Hospital Healthy Heart Program. The nutrition, exercise, and cardiac rehab sessions are described in detail in the Supplemental Methods.

#### Part 1—nutrition (months 1 to 4)

A 4-month program was led by a registered dietitian in a class size of approximately 20 participants. The nutrition program consisted of 2 individual nutrition assessments with a dietician, 6 group-based cognitive behavioural therapy sessions, and 6 group-based nutrition education and food skills sessions. Sessions were held at Garratt Wellness Centre, a community health partnership between the City of Richmond and Vancouver Coastal Health—Richmond Health Services. The nutrition education and food skills sessions included dietary goal setting, nutrition label-reading, meal planning, and 3 hands-on kitchen-based practical skills sessions. Description of each cognitive behavioural therapy and nutrition session can be found in the Supplemental Methods. Meal supplements were not used or recommended. Participants were not required to report their weight. The classes were nonjudgmental and noncompetitive. Group discussion was encouraged, to build camaraderie, address challenges related to lifestyle change, and positively reinforce healthy dietary habits.

#### Part 2—home-based exercise (months 1 to 6)

At the start of the intervention, participants in the intervention group received a 30-minute individualized, in-person consultation with an exercise health coach (certified kinesiologist) and received a personalized home exercise program for months 1 to 4 of the intervention. The 4-month home exercise program started with three 20-minute sessions of physical activity per week, and progressed to five 40-minute sessions of physical activity per week (a total of 200 min/wk). The consultation also taught participants how to monitor their heart rate and keep within their individualized target heart rate zone using the Fitbit. Each participant was given an exercise log sheet to record the type, intensity, and duration of exercise, along with their peak heart rate each week. At the weekly nutrition classes, participants handed in their exercise log sheets to encourage adherence. If any issues with their Fitbit occurred, these were addressed in the nutrition classes.

#### Part 3—supervised exercise and education (months 4 to 6)

Cardiac rehabilitation (CR) took place twice weekly during months 4 to 6 and included moderate-intensity interval training at the Richmond Hospital Healthy Heart Program. Before starting in-center exercise, each participant received an individualized 30-minute consultation with the CR nurse and CR physiotherapist who ran the program and who were present at all classes to determine individual goals and limitations. In addition, volunteer university students helped participants track their exercise intensity and duration and ensured a 2:1 ratio of participants to staff. Polar chest strap heart rate monitors (Polar Electro, Kempele, Finland) were used for all participants during in-house exercise. Each cohort of 20 was divided into groups of 10 for exercise sessions. The curriculum included 3 nutrition classes (in addition to those described above), 4 stress-management classes, and 1 pharmacy education class.

The second phase (phase 2) consisted of 6 months of home-based maintenance exercise. Participants were encouraged to continue to apply the nutrition principles, based on Canada’s Food Guide, and practice the nutrition skills and behaviour-modification techniques learned during the 4-month nutrition curriculum.

All intervention participants whose glycosylated hemoglobin (HbA1C), blood pressure (BP), and/or lipids were uncontrolled^19^, ^20^, ^21^ were referred to an internist for risk-factor management. Details on referral criteria, interventions, and targets are included in the Supplemental Methods.

### Control group

In addition to receiving a Fitbit, guideline-based AF care, and instructions on Canada’s Food Guide and Physical Activity Guidelines, the control participants also received handouts including weight-loss tips and recommendations for exercises that are safe for individuals with AF to do at home. Patients otherwise received “usual care” from their primary cardiologist.

### Data collection

Baseline testing occurred between 0- and 3-months pre-randomization and included assessment of cardiovascular risk factors (Hb A1c, total cholesterol, low-density lipoprotein, high-density lipoprotein, triglycerides), an electrocardiogram, and assessment of fitness measured as metabolic equivalents of tasks (METs) on a symptom-limited exercise stress test. The physical exam included measurements of height, weight, BMI, abdominal circumference, and neck circumference, a cardiovascular physical exam, and systolic and diastolic blood pressure measured while participants were seated after 5 minutes of rest with an Omron 902 automated office BP monitor. Baseline medical history was taken by the enrolling cardiologist and included emergency department visits for documented AF, electrical cardioversions, doctor-reported burden of AF, and medications. Medication use (antiarrhythmic agents, antihypertensive agents, diabetes and cholesterol-lowering medications) was documented.

#### AF burden

Paroxysmal AF is defined as a continuous AF episode lasting longer than 30 seconds but terminating spontaneously or with intervention within 7 days of onset. Persistent AF is defined as continuous AF episodes lasting longer than 7 days, but less than 1 year. In participants with both paroxysmal and persistent AF, the predominant pattern was recorded. Doctor-reported burden of AF (herein referred to as “DR burden”) was defined using the following scale:

1 = none (no documented AF in 12 months);
2 = existing AF (AF documented in 12 months);
3 = low burden (paroxysmal AF: ≥ 2 episodes of AF over the past 24 months; episodes terminate spontaneously within 7 days or via cardioversion within 48 hours of onset);
4 = high burden (paroxysmal AF ≥ 4 episodes of AF over the past 6 months, with ≥ 2 episodes > 6 hours in duration; episodes terminate spontaneously within 7 days or via cardioversion within 48 hours of onset).

The burden of AF was also measured using a 48-hour Holter monitor, the doctor-reported Canadian Cardiovascular Society Severity of Atrial Fibrillation Scale (CCS-SAF),^22^ and the 19-item self-report University of Toronto Atrial Fibrillation Symptom Severity Scale (AFSS).^23^ The CCS-SAF quantifies the functional illness burden of AF into classes 0 to 4. The AFSS is a disease-specific QoL measure scored for AF symptoms, duration, severity, and a sum score representing total AF burden.

#### Mental health and QoL outcomes

QoL was measured using the 36-item Short Form Survey Instrument (SF-36), which includes 8 subscales.^24^ We also calculated the aggregate Physical Component Score (PCS) and Mental Component Score (MCS), using normative data.^25^ Anxiety and depression were measured using the Generalized Anxiety Disorder 7-item (GAD-7)^26^ and the 8-item Personal Health Questionnaire (PHQ-8)^27^

#### Sleep apnea

All patients were screened for sleep apnea using the Epworth sleepiness scale (ESS).^28^ A baseline-level 3-sleep study was performed on all participants without a sleep study in the last 2 years, regardless of ESS. All participants with moderate or severe sleep apnea (Apnea-Hypopnea Index > 15), or mild sleep apnea (index > 5) with daytime sleepiness, were offered a consultation with an internist who specializes in sleep medicine. Participants already using continuous positive airway pressure were not re-tested but were asked to check with their sleep medicine specialist to optimize therapy.

#### Echocardiogram

One technician performed all echocardiograms using a Phillips IE33 (Andover, MA), and one cardiologist read all images, obtaining measurements per Lang et al.^29^ Both were blinded as to participant allocation.

At 6 months, all baseline testing was repeated, with the exception of the echocardiogram and the level-3 sleep study. At 12 months, cohort 1 repeated baseline testing, with the exception of the level-3 sleep study. Due to COVID-19 pandemic restrictions, the 12-month follow-up for cohort 2 was limited to QoL, PHQ-8, and GAD-7 conducted via surveymonkey.com (online) or via mailed-out surveys. A cardiologist took a 12-month follow-up medical history for cohort 2 as a virtual follow-up with patient consent.

### Outcomes

Planned co-primary outcomes were as follows: (i) percent time in AF on a 48-hour Holter monitor; (ii) severity of AF on the CCS-SAF and AFSS scales; and (iii) change in QoL on the SF-36 at 6 and 12 months.

Due to limitations related to the COVID-19 pandemic, we were not able to perform Holter monitoring or in-person assessment of AF burden and symptoms for cohort 2 at 12 months. We therefore limited our primary outcome to QoL as measured by SF-36 at 6 and 12 months.

Secondary outcomes included changes from baseline to 6 months for the following: percent AF beats on 48-hour Holter monitor; CCS-SAF score; AFSS score; mental health scores (GAD-7, PHQ-8); cardiovascular risk factors (BP, HbA1c, total cholesterol, high-density lipoprotein, low-density lipoprotein, triglycerides, BMI, waist circumference); fitness (METs achieved during stress test); medications (amiodarone use, other antiarrhythmic agent use, antihypertensive agent use); number of hospital visits for irregular rhythm; number of cardioversions; and ablation.

We reassessed the above secondary outcomes and echocardiogram findings at 12 months for patients in cohort 1, but this was not possible for cohort 2, due to the COVID-19 pandemic.

### Sample size

Using G-power software (Heinrich-Heine-Universität Düsseldorf, Düsseldorf, Germany) (alpha = 0.05), the sample size required to achieve 80% power to detect an effect is 21 participants per group (using analysis of covariance [ANCOVA]). Calculation of sample size was done *a priori* during trial design, based on large effect sizes (*f* = 0.5) reported by Malmo and colleagues^6^ for the difference in time spent in AF pre- and post-exercise. When removing AF burden as our primary outcome, we kept our *a priori* power calculation, as the trial had already begun, and for transparency in our trial design.

### Statistical analysis

Normally distributed data were analyzed using ANCOVA to compare the effect of the intervention while taking baseline data into account for each group. Follow-up analyses on differences from baseline were analyzed using paired (dependent) *t*-tests. Statistical significance was set *a priori* as *P* < 0.05.

## Results

### Study participants and baseline demographics

**Figure 1:**
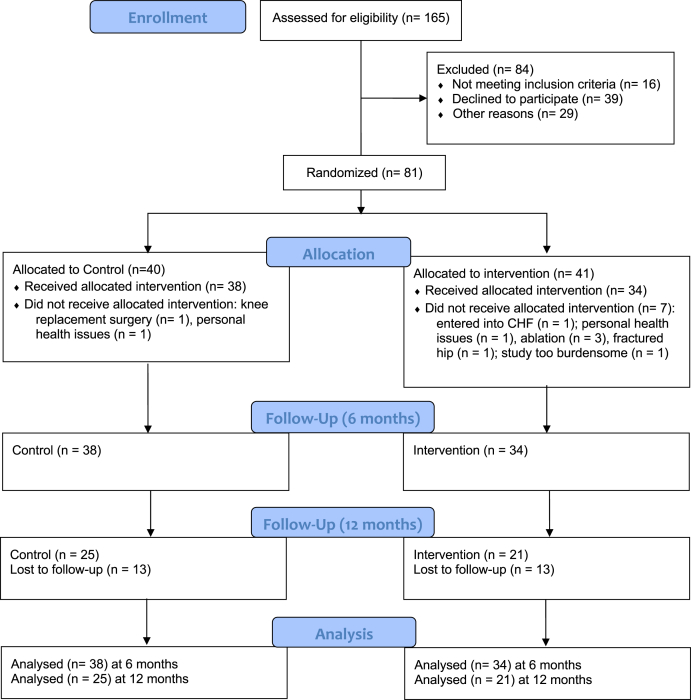
Flowchart of the screening process, showing how many participants were screened and how many were excluded from the study, along with the number of participants that were used for the final analysis. CHF, congestive heart failure.

Of the 165 consecutive patients with AF screened over 6 months (January to June 2018), 149 patients met eligibility criteria (Fig. 1). A total of 68 patients declined to participate due to the required time commitment, travel considerations, or interest level, and the remaining 81 participants were randomized into either the control group (n = 40) or the intervention group (n = 41) in 2 cohorts run in series (August 2018 to February 2019; January to June 2019). Following randomization, 9 participants dropped out of the study, resulting in a final sample size of 72 (mean age 62 ± 9.2 years, 61% male; control n = 38; intervention n = 34).

The groups did not exhibit any significant differences in baseline characteristics (all *P* > 0.05; Table 1). Our sample comprised individuals self-reporting as being European (62.5%), East Asian (16.7%), South Asian (6.9%), South East Asian (4.2%), and Latin American (2.8%), and 6.9% who chose not to provide information on race/ethnicity.

**Table 1:** Participant demographics at baseline

Characteristic	Control (n = 38)	Intervention (n = 34)
Demographic features		
Age, y	61.0 ± 9.7	63.7 ± 8.6
Male sex	21 (55.3)	23 (67.7)
BMI, kg/m^2^	31.3 ± 5.5	31.5 ± 5.4
Waist circumference, cm	105.4 ± 12.5	107.6 ± 13.0
High-risk waist circumference∗	32 (84.2)	31 (91.2)
SBP, mm Hg	123.0 ± 14.6	125.2 ± 18.8
Type of AF		
Persistent	3 (7.9)	5 (14.7)
Paroxysmal	35 (92.1)	29 (85.3)
DR burden = low or high†	14 (36.8)	17 (50)
DR burden = none or existing†	24 (63.2)	17 (50)
Comorbidities		
Diabetes	8 (21.1)	7 (20.6)
Hypertension	23 (60.5)	23 (65.7)
Coronary artery disease	5 (13.2)	5 (14.7)
Stroke	3 (7.9)	4 (11.8)
Sleep apnea	27 (71.1)	23 (67.6)
Smoking (> 0 pack years)	16 (42.1)	11 (32.4)
Alcohol (> 3 servings/d)	2 (5.3)	5 (14.7)
Alcohol (> 7 servings/wk)	8 (21.1)	6 (17.6)
Treatment and medication		
Previous ablation	5 (13.2)	5 (14.7)
Cardioversion (past 6 mo)	8 (21.1)	13 (38.2)
Amiodarone	2 (5.3)	5 (14.7)
Other anti-arrhythmic agent(s)	10 (26.3)	14 (41.2)
Beta-blocker	16 (42.1)	22 (64.7)
Other hypertension medication(s)	24 (63.2)	21 (61.8)
Diabetes medication(s)	9 (23.7)	4 (11.8)
Echocardiographic measures		
LA volume index, ml/m^2^	37.1 ± 8.35	37.9 ± 8.7
LVEF, %	60.7 ± 4.82	61.4 ± 4.3
LV mass index, g/m^2^	79.5 ± 15.7	81.7 ± 14.4

Values are n (%) or mean ± standard deviation. To determine baseline comparisons between groups, the independent *t*-test was used for continuous variables and the χ^2^ test was used for frequencies. *P* < 0.05 was considered significant; there were no significant differences between groups.

AF, atrial fibrillation; BMI, body mass index; DR, doctor-reported; LA, left atrium; LV, left ventricle; LVEF, left ventricular ejection fraction; SBP, systolic blood pressure.

∗ Asians ≥80 cm for females and ≥90 cm for males; Caucasians ≥88 cm for females and ≥102 cm for males.

† DR burden: none = no documented AF in 12 months; existing = AF documented in 12 months; low = 2 or more episodes of AF in 24 months; high = 4 or more episodes of AF in 6 months with 2 or more lasting > 6 hours.

### Echocardiography baseline data

At baseline, the mean left atrial volumes indexed per body surface area were mildly increased (> 34 cc/m^2^) in both the intervention and control groups. The left ventricular ejection fraction and left ventricular mass were normal. Additional baseline and 12-month echocardiographic parameters are reported in Supplemental Table S1.

### Primary outcomes

**Figure 2:**
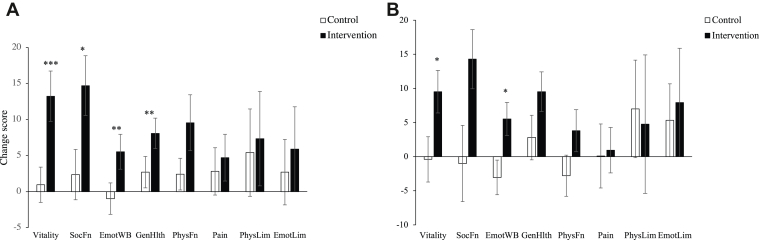
Change scores for 36-item Short Form Survey Instrument subscales from (**A**) baseline to 6 months and (**B**) baseline to 12 months (n = 25 for control; n = 21 for intervention). ∗*P* < 0.05; ∗∗*P* < 0.01; ∗∗∗*P* < 0.001 for difference between intervention and control groups. **Error bars** represent standard error of change score. SocFn, social functioning; EmotWB, emotional well-being; GenHlth, general health; PhysFn, physical functioning; Pain, bodily pain; PhysLim, role limitations due to physical health; EmotLim, role limitations due to emotional health.

Data were screened for normality, and assumptions for analysis of covariance (ANCOVA) were satisfied. The intervention resulted in significant improvements in QoL as measured by the SF-36 at 6 months (Fig. 2A; Table 2). The intervention group improved, compared with the control group, in the following SF-36 subscales: vitality (*P* < 0.001), social functioning (*P* = 0.018), emotional well-being (*P* = 0.017), and general health perceptions (*P* = 0.009), and on the mental component score (*P* = 0.024). Additionally, follow-up, paired *t*-tests revealed that the intervention group reported an improvement in physical functioning (*P* = 0.019), whereas the control group did not change (*P* = 0.28). The intervention had no significant effects on the remaining subscales—bodily pain, role limitations due to physical problems, role limitations due to emotional problems (*P* > 0.05)—or on the physical component score. At the 12-month follow-up, improvement in the SF-36 subscales of vitality (*P* = 0.021) and emotional well-being (*P* = 0.036) remained significant (Fig. 2B).

**Table 2:** Quality of life (SF-36) at baseline and 6 months

Outcome	C group (n = 37)		*P*∗	I group (n = 34)		*P*∗	*P*†
	Baseline	6 mo		Baseline	6 mo		Between groups
Vitality	54.3 ± 20.6	55.3 ± 19.0	0.702	57.2 ± 18.7	70.4 ± 14.6	0.001	0.000‡
Social functioning	76.4 ± 23.0	78.7 ± 26.5	0.502	74.3 ± 21.5	89.0 ± 15.3	0.001	0.018§
Emotional well-being	72.5 ±19.0	71.6 ± 19.3	0.659	74.0 ± 15.4	79.5 ± 11.9	0.029	0.017§
General health	56.8 ± 18.0	59.5 ± 17.8	0.223	62.5 ± 14.6	70.6 ± 12.5	0.274	.009‖
Physical functioning	76.9 ± 22.8	79.3 ± 23.2	0.276	70.4 ± 25.3	80.0 ± 19.4	0.019	0.233
Pain	73.0 ± 25.4	75.9 ± 24.3	0.397	74.0 ± 21.7	78.7 ± 22.3	0.156	0.599
Limitations, due to physical factors	62.2 ± 38.9	67.6 ± 38.6	0.378	65.4 ± 38.0	72.8 ± 33.9	0.270	0.628
Limitations, due to emotional factors	73.0 ± 37.6	75.7 ± 37.4	0.556	75.5 ± 36.1	81.4 ± 24.9	0.324	0.476
Physical component score	46.0 ± 10.1	47.9 ± 10.9	0.114	45.4 ± 10.5	48.7 ±10.4	0.025	0.473
Mental component score	41.1 ± 16.7	41.2 ± 17.0	0.992	42.5 ± 16.1	47.7 ± 10.5	0.028	0.024§

Values are mean ± standard deviation, unless otherwise noted.

C, control; I, intervention.

∗ *P* value for the within group differences (baseline to 6 months).

† *P* value for the between-group differences over time (analysis of co-variance with baseline characteristics as covariate); missing 6 month data for 1 control participant.

‡ *P* < 0.001.

§ *P* < 0.05.

‖ *P* < 0.01; for difference between intervention and control groups.

### Secondary outcomes

The intervention led to a significant reduction in the severity of AF as measured by the doctor-reported CCS-SAF score (*P* = 0.01; Table 3), but not in the frequency of AF as measured by a 48-hour Holter monitor at 6 months (*P* = 0.469) or the self-reported AFSS frequency scale (*P* > 0.05).

**Table 3:** Atrial fibrillation (AF) burden at baseline and 6 months

Outcome	C group (n = 38)		*P*∗	I group (n = 34)		*P*∗	*P*†	Missing data
	Baseline	6 mo		Baseline	6 mo		Between groups	(C/I)
AF frequency								
% AF beats, 48-h Holter monitor	8 ± 21	4.8 ± 17.6	0.668	2.2 ± 11.5	8.2 ± 22.1	0.199	0.469	2/3
Holter N < 1 % AF	30 (78.9)	32 (88.9)	0.453	31 (91.2)	25 (80.6)	0.250	0.126	—
Holter N > 5 % AF	8 (21.1)	4 (11.1)	0.453	3 (8.8)	6 (19.4)	0.250	0.126	—
AF burden								
DR burden	2.3 ± 1.0	2.1 ± 0.9	0.132	2.6 ± 1.0	2.3 ± 1.0	0.014	0.921	1/1
CCS-SAF score	1.5 ± 1.0	1.4 ± 1.1	0.672	1.5 ± 1.1	0.9 ±1.1	0.001	**0.010**	0/0
AFSS score								
Frequency	2.9 ± 2.7	2.7 ± 2.5	0.688	2.8 ± 2.5	2.4 ± 2.4	0.418	0.616	1/0
Duration	3.2 ± 2.7	3.6 ± 3.0	0.254	3.1 ± 2.9	3.0 ± 2.9	0.261	0.230	1/0
Severity	5.4 ± 3.1	4.8 ± 2.7	0.034	4.4 ± 2.7	4.7 ± 2.4	0.468	0.278	1/0
Symptom	8.1 ± 6.7	5.4 ± 5.2	0.007	7.8 ± 5.2	5.4 ± 4.9	0.008	0.875	1/0
Global WB	7.1 ± 1.9	11.2 ± 5.6	368	10.3 ± 5.9	10.0 ± 5.6	0.524	0.739	1/1

Values are mean ± standard deviation, or n (%), unless otherwise noted. Boldface indicates significance.

AFSS, Atrial Fibrillation Symptom Severity Scale; C, control; CCS-SAF, Canadian Cardiovascular Society Severity of Atrial Fibrillation Scale; DR, doctor-reported; I, intervention; WB, well-being.

∗ *P* value for the within-group differences (baseline to 6 months);

† *P* value for the between-group differences over time (analysis of covariance with baseline characteristics as covariate).

Both groups also showed minimal weight loss at 6 months (control, 91.5 kg vs 89.5 kg, *P* = 0.02; intervention, 91.1 kg vs 89.6 kg, *P* = 0.03), and waist circumference in the intervention group decreased slightly (*P* = 0.04), with no intervention effect (*P* > 0.05; Table 4). The intervention had no significant effects on other physiological and anthropometric parameters (all *P* > 0.05) or in follow-up pairwise tests comparing 6-month to baseline data. Results for 12 months are similar (Supplemental Table S2).

**Table 4:** Secondary outcomes at 6 months

Outcome	C group (n = 38)		*P*∗	I group (n = 34)		*P*∗	*P*†	Missing data
	Baseline	6 mo		Baseline	6 mo		Between groups	(C/I)
BMI, kg/m^2^	31.3 ± 5.5	31.0 ± 5.5	0.255	31.5 ± 5.4	31.2 ± 5.2	0.191	0.838	—
Waist, cm	105.4 ± 12.5	101.8 ± 19.6	0.294	107.6 ± 13.0	104.8 ± 12.4	0.004	0.645	—
Weight, kg	91.5 ± 19.7	89.5 ± 19.8	0.024	91.1 ± 18.2	89.6 ± 17.6	0.025	0.630	—
SBP, mm Hg	123 ± 14.6	119.7 ± 13	0.089	125.2 ± 18.8	121.6 ± 17.0	0.205	0.784	—
DBP, mm Hg	72.5 ± 11.5	73 ± 15.5	0.849	72.2 ± 9.8	70.2 ± 10.0	0.223	0.374	—
Peak METs	8.7 ± 2.8	9.5 ± 3.2	0.000	9.0 ± 3.5	9.9 ± 3.8	0.001	0.649	—
Lipid status								
Total cholesterol	4.2 ± 0.9	4.1 ± 1.0	0.637	4.3 ± 1.1	4.3 ± 1.1	0.891	0.522	4/2
LDL	2.2 ± 0.9	2.1 ± 0.9	0.523	2.2 ± 0.9	2.2 ± 0.9	0.786	0.565	6/3
HDL	1.3 ± 0.4	1.4 ± 0.4	0.212	1.5 ± 0.4	1.6 ± 0.5	0.088	0.572	4/2
Triglycerides	1.7 ± 1.6	1.4 ± 0.7	0.404	1.8 ± 2.3	1.3 ± 0.9	0.154	0.267	4/2
HbAIC	6.1 ± 1	5.9 ± 0.6	0.269	6.1 ± 1.5	5.9 ± 1.1	0.111	0.644	4/2
Hospital visits								
ER visits‡	12 (31.6)	7 (18.4)	0.227§	9 (26.5)	3 (8.8)	0.109§	0.277‖	0/1
Ablation	0 (0)	0 (0)	1.00	0 (0)	0 (0)	1.00	1.00	0/0
Cardioversion	8 (21.1)	4 (10.5)	0.289§	13 (38.2)	3 (8.8)	0.006§	0.565	0/0
Medications								—
Amiodarone	2 (5.3)	2 (5.3)	1.00	5 (14.7)	5 (14.7)	1.00	1.00	—
Anti-arrhythmic agent(s)	10 (26.3)	14 (36.8)	0.125	14 (41.2)	12 (35.3)	0.625	0.080	—
Hypertension medication(s)	24 (63.2)	24 (63.2)	1.0	21 (61.8)	24 (70.6)	0.250	0.147	—
Mental health								
GAD-7	3.8 ± 4.7	3.9 ± 5.0	0.626	4.1 ± 4.7	3.0 ± 3.4	0.100	0.154	1/0
PHQ-8	4.8 ± 5.2	4.1 ± 4.3	0.365	4.2 ± 4.9	3.0 ± 2.7	0.064	0.179	1/0

Values are mean ± standard deviation, or n (%), unless otherwise noted.

BMI, body mass index; C, control; DBP, diastolic blood pressure; ER, emergency room; GAD-7, Generalized Anxiety Disorder 7-item; HbA1c, glycosylated hemoglobin; HDL, high-density lipoprotein; I, intervention; LDL, low-density lipoprotein; MET, metabolic equivalent of tasks; PHQ-8, 8-item Personal Health Questionnaire.

∗ *P*-value for the within-group differences (baseline to 6 months).

† *P*-value for the between-group differences over time (analysis of covariance with baseline characteristics as covariate).

‡ ER visits for an irregular heart rhythm.

§ *P*-value obtained using McNemar test for binary variables.

‖ Homogeneity of variance assumption violated (significant Levene’s test).

Improvements were seen in fitness, as measured by METs, in both the control (*P* < 0.001) and intervention groups (*P* = 0.001), with no effect of the intervention (*P* > 0.05).

The ESS score significantly decreased in the intervention group, relative to that in the control group (*P* = 0.04). At baseline, 84% of our patient population had sleep apnea. Follow-up tests revealed that the ESS score in the intervention group decreased, whereas the scores in the control group increased, although neither pairwise comparisons from baseline were significant (*P* = 0.7).

Although we observed changes in mental health outcomes related to QoL (described above), no significant differences between groups occurred in the other mental health indicators, including the GAD-7 scale for anxiety and the PHQ-8 for depression (*P* > 0.05).

Finally, the intervention did not significantly affect the number of emergency department visits or ablations. However, a reduction from baseline occurred in the number of cardioversions in the intervention group (*P* = 0.006), but the difference was not significant relative to the control group (*P* = 0.565).

## Discussion

This randomized controlled trial studied the impact of a 6-month, intensive, supervised medical optimization, nutrition, exercise, and stress-management program, followed by a 6-month maintenance program, compared to usual care. We found that the intensive program significantly improved QoL at 6 months, with some improvements persisting through the self-directed maintenance phase to 12 months, despite there being no decrease in AF burden at 6 months.

### QoL

AF is associated with significant reduction in QoL,^2^ and current treatment strategies may contribute to decreased QoL.^30^ In this study, improvements in QoL were seen in both the intervention and control groups across most domains of the SF-36 at 6 months, with significantly greater improvement in the intervention group in the areas of vitality, social function, emotional well-being, and general health. At 12 months, the improvement in QoL persisted for vitality and emotional well-being. Although the SF-36 was not specifically developed for assessment of AF-specific QoL, its use is recognized in guidelines, along with its advantage of having been validated across diverse populations and multiple medical conditions.^1^ Additionally, the SF-36 was used to validate disease-specific tools, including the CCS-SAF score.^22^ A meta-analysis of exercise interventions in AF patients similarly reported improvement in vitality and general health subscales on the SF-36.^10^ Previous trials of weight loss in those with AF have not examined the effect on QoL.^11^, ^12^, ^13^ Although we did not conduct a qualitative analysis of the factors that led to the observed QoL, we speculate that enhanced social interactions and interpersonal support in class and during exercise, increased communication with physicians and allied healthcare team members, and the establishment of long-term, healthy active-living routines played a role.

### AF burden

A reduction in AF burden occurred, as measured by the doctor-reported CCS-SAF score, but no reduction occurred in AF burden, as measured by Holter or AFSS score. Instead, a trend toward decreased AF burden occurred in the control group, and a trend toward an increased AF burden occurred in the intervention group. In contrast, previous small studies have shown a reduction in AF burden with weight loss^12^^,^^13^ and exercise interventions.^6^^,^^31^ Weight loss and exercise recommendations have been included in the 2020 Canadian AF Guidelines, which include an ungraded note targeting a BMI < 27 kg/m^2^ and 10% body-weight loss.^1^ However, no recommendation is given for how to achieve this weight loss, and Obesity Canada’s guidelines do not offer any dietary strategy that is capable of sustaining this degree of weight loss.^32^

In contrast to previous studies that specifically assess weight loss or exercise interventions, patients in our study did not have significant weight loss and showed only slight improvement in exercise capacity. Similarly, the intervention group did not show significant improvement in metabolic parameters associated with weight loss and exercise, such as BP or HbA1c. Studies that have shown that weight loss resulted in reduction in AF burden have utilized extreme caloric restriction and meal replacements and/or at least twice as many individualized weight-loss counselling sessions.^11^, ^12^, ^13^ Our nutrition intervention provided group educational sessions and did not recommend liquid meal replacements, caloric or macronutrient restrictions, nor did it eliminate food groups or enforce regular weigh-ins.

### Cardiovascular outcomes

The intervention did not significantly affect clinical or cardiovascular outcomes, including the number of emergency department visits, cardioversions, and ablations. Previous studies of exercise and CR similarly have found no significant difference in hospitalization rate or cardioversions.^33^^,^^34^ although Malmo and colleagues did find a trend in this direction.^6^ In contrast, a comprehensive mobile health intervention, which included a focus on underlying conditions, showed improvement in cardiovascular outcomes; however, the comprehensive intervention was associated with greater use of anticoagulants, antiarrhythmics, beta-blockers, angiotensin-converting enzyme inhibitors/angiotensin receptor blockers, and statin,^35^ which are known to improve outcomes. Another comprehensive study targeting underlying conditions in those with AF did not show significant reduction in composite cardiovascular outcome, hospitalizations, or other adverse events.^36^

### Limitations

Cardiovascular risk factors, including BP, HbA1c level, and weight, did not significantly improve, despite the lifestyle intervention. Patients in our trial were relatively well at baseline, with lower baseline BP and BMI, relative to those in participants in other studies.^6^^,^^12^^,^^13^^,^^31^^,^^36^ Also, our intervention did not result in significant weight loss, which was seen in trials that showed improvement in AF burden.^6^^,^^12^ However, these studies were either nonrandomized or employed meal-replacement shakes, which we chose to avoid. Sustained weight loss of the degree that has shown benefit in previous trials may not be realistic,^32^ and the addition of pharmacologic weight-loss treatment represents an area for future study.

Additionally, we used a 48-hour Holter monitor to assess AF burden at baseline and follow-up, whereas other trials used 7-day monitors or implanted loop recorders. A 48-hour measurement can underestimate or overestimate the true burden of AF.

Finally, the COVID-19 pandemic limited our follow-up. Due to public health restrictions, we were unable to conduct in-person assessment or echocardiogram at 12 months for cohort 2. We attempted virtual follow-up, and used mailed and online surveys, but we had a greater-than-expected number of dropouts. The high number of dropouts caused loss of power for assessing QoL at 12 months, and significance was lost, despite a similar trend in QoL outcomes.

## Conclusion

Overall, this study demonstrated that a supervised group CR and nutrition program improved QoL, compared with a self-directed program, despite no improvement in AF burden or cardiovascular risk factors. Limitations included well-controlled cardiovascular risk factors at baseline and a lack of robust monitoring for AF burden.
