# Supplementary material for: Effect of an Exercise and Nutrition Program on Quality of Life in Patients With Atrial Fibrillation: The Atrial Fibrillation Lifestyle Project (ALP)
Source: CJC Open. 2022 Apr 27;4(8):685–94. doi: 10.1016/j.cjco.2022.04.004 (PMC9402963; doi:10.1016/j.cjco.2022.04.004)
Supplement: Supplemental Material [file mmc1.pdf]

Supplementary Material

Supplemental Tables

- Supplemental Table S1. Echocardiogram results at baseline and 12 months.
- Supplemental Table S2: Outcomes at 12 months

Supplemental Methods:

- Nutrition intervention
- Exercise intervention
- Comorbidity referral and targets.

Supplemental Tables

Supplemental Table S1. Echocardiogram results at baseline and 12 months.

Parameter	Control		Intervention	
	Baseline (*n* = 38)	12-months (*n* =14)	Baseline (*n* = 34)	12-months (*n* = 14)
LA volume index (ml/m^2)	37.1 ± 8.4	37.2 ± 9.4	37.9 ± 8.7	37.0 ± 8.6
LVEF	60.7 ± 4.8	63 ± 3.9	61.4 ± 4.3	60.4 ± 3.5
LV mass	79.5 ± 15.7	75.7 ± 18.5	81.21 ± 14.2	81.2 ± 12.5
IVSd (mm)	9.7 ± 1.1	9.5 ± 0.7	9.72 ± 1.3	939 ± 0.9
LVIDd (mm)	46.6 ± 4.6	44.7 ± 8.8	47.1 ± 4.8	43.6 ± 9.4
Average E/e’ ratio	9 ± 2.9	9.5 ± 2.8	9.3 ± 2.8	9.9 ± 3.2
LV GLS	-17.7 ± 3.4	-18.5 ± 2.9	-18.4 ± 3.7	-17.8 ± 3.0

Note. The echocardiogram data at 12 months was only obtained for cohort 1. The 12-month followup for cohort 2 coincided with COVID-19 pandemic and all unnecessary in-person follow-ups were cancelled. There were no significant differences between groups over time.

LA, left atrium; LVEF, left ventricular ejection fraction; LV, left ventricle; IVSd, interventricular septal thickness at end diastole; LVIDd, left ventricular internal diameter in diastole; E/e’, early mitral inflow velocity /early diastolic mitral annular velocity; LV GLS, left ventricular global longitudinal strain

Supplemental Table S2: Outcomes at 12 months

Outcome	Control (C) (*n* =)		p^a *value*	Intervention (I) (*n* =)		p^a *value*	p^b *value*	Observed data
	Baseline	12 months		Baseline	12 months		Between groups	(C/I)
BMI, kg/m^2	31.3 ± 5.5	31.4 ± 5.3	.468	31.5 ± 5.4	30.0 ± 5.0	.002	.363	9/6
Waist, cm	105.4 ± 12.5	104.9 ± 9.7	.853	107.6 ± 13.0	102.4 ± 12.1	.278	.226	9/6
Weight, kg	91.5 ± 19.7	101.4 ± 27.2	.585	91.1 ± 18.2	83.9 ± 17.9	.012	.313	9/6
SBP, mmHg	123 ± 14.6	140.1 ± 11.9	.125	125.2 ± 18.8	134.7 ± 9.4	1.00	.499	9/6
DBP, mmHg	72.5 ± 11.5	80.4 ± 6.6	.524	72.2 ± 9.8	72.8 ± 10.5	.739	.252	9/6
Peak METs	8.7 ± 2.8	12.6 ± 2.0	.497	9.0 ± 3.5	9.5 ± 3.1	.009	.213	4/6
Epworth Sleep Score	5.6 ± 3.4	6.2 ± 5.3	.639	4.6 ± 2.8	4.9 ± 4.2	.820	.645	14/13
On CPAP, *n*(%)	8 (21.1)	7 (29.2)	.250	10(29.4)	8(38.1)	1.00	.715	14/13
AF burden								
AF burden 1	2.3 ± 1.0			2.6 ± 1.0				
CCS-SAF score	1.5 ± 1.0	1.2 ± 1.2	.274	1.5 ± 1.1				
AFSS								
AF frequency (range 1-10)	2.9 ± 2.7	3.5 ± 2.7	.047	2.8 ± 2.5	2.9 ± 2.3	.440	.391	25/21
AF duration (range 1-10)	3.2 ± 2.7	2.9 ± 2.5	.161	3.1 ± 2.9	2.7 ± 2.3	.196	.290	25/21
AF severity (range 1-10)	5.4 ± 3.1	4.2 ± 3.0	.232	4.4 ± 2.7	4.1 ± 2.5	.471	.832	25/21
Global WB (range 1-10)	7.1 ± 1.9	7.7 ± 1.9	.310	7.2 ± 1.8	7.6 ± 1.8	.171	.967	25/21
AF symptom (range 1-35)	8.1 ± 6.7	5.6 ± 5.4	.454	7.8 ± 5.2	4.3 ± 3.7	.006	.191	25/21
Hospital visits								
ER visits *n*(%)	12 (31.6)	4 (14.3)	.388	9 (26.5)				28/23

Ablation *n*(%)	0 (0)	0 (0)	1.00	0 (0)	0 (0)	1.00	-	-
Cardioversion *n*(%)	8 (21.1)	0 (0)	.062	13 (38.2)				27/23
Mental health								
GAD-7	3.8 ± 4.7	3.2 ± 5.9	.679	4.1 ± 4.7	3.6 ± 4.7	.477	.628	13/13
PHQ-8	4.8 ± 5.2	3.4 ± 4.3	.091	4.2 ± 4.9	2.2 ± 1.5	.309	.086	18/20

Note. Data requiring in-person assessments at 12 months was only obtained for cohort 1. The 12-month follow-up for cohort 2 coincided with COVID-19 pandemic and all unnecessary in-person follow-ups were cancelled.

BMI, body mass index; SBP, systolic blood pressure; DBP, diastolic blood pressure; MET, metabolic equivalent of tasks; CPAP, continuous positive airway pressure; AF, atrial fibrillation; CCS-SAF, Canadian Cardiovascular Society Severity of Atrial Fibrillation Scale; AFSS, Atrial Fibrillation Symptom Severity Scale; WB, wellbeing; ER, emergency room; GAD-7 Generalized Anxiety Disorder 7-item; PHQ-8, 8-item Personal Health Questionnaire.

Supplemental Methods

Nutrition intervention

Nutrition: A four-month nutrition program (months one to four) was led by a registered dietitian in a class size of approximately 20 participants. The nutrition program consisted of two individual nutrition assessments with a dietician, six group-based cognitive behaviour therapy (CBT) sessions, and six group-based nutrition education and food skills sessions. Sessions were held at Garratt Wellness Centre, a community health partnership between the City of Richmond and Vancouver Coastal Health – Richmond Health Services. The CBT sessions were designed to help participants identify their eating triggers and manage food cravings, which included weekly assignments and setting goals. The nutrition curriculum modelled Canada's Food Guide emphasizing high-fibre vegetables and fruits, lean protein and minimally processed foods[29]. The nutrition education and food skills sessions included dietary goal setting, nutrition label-reading, meal planning, and three hands-on kitchen-based practical skills sessions. Description of each CBT and nutrition session can be found in supplemental data. Meal supplements were not used or recommended. Participants were not required to report their weight. The classes were non-judgmental or competitive, but

supportive, and group discussion was encouraged to build camaraderie, address challenges related to lifestyle change, and positively reinforce healthy dietary habits.

Nutrition Curriculum Content

Nutrition Class 1: Defining your nutrition and weight loss goals

- Practical skill: SMART goal setting framework to define short (i.e. this week), medium (i.e. this month) and long term (i.e. this year and beyond) goals

Nutrition Class 2: The basics of calories, macronutrients and micronutrients

- Practical skill: How to read a nutrition facts label and ingredient list

Nutrition Class 3: Hands-on carbohydrates

- Practical skill: Understanding "serving size" versus portion size and how to optimize the nutrient intake by choosing high-fiber, low-sugar options

Nutrition Class 4: Hands-on proteins and fats

- Practical skill: Understanding "serving size" versus portion size and how to optimize the nutrient intake by choosing lean protein, poly- and mono-unsaturated fats

Nutrition Class 5: Building well-balanced meals and snacks

- Meal planning using the healthy plate model (Canada's Food Guide) and learning food preparation skills to maximize opportunities for healthy eating

Nutrition Class 6: Healthy dining when eating out, while on vacation and during holidays

- Practical skills: Strategies to avoid overeating and the "all-or-nothing" mindset

Cognitive Behaviour Therapy Curriculum

Cognitive Behaviour Therapy Class 1: The fundamentals of balanced meals

- How to improve what, when and how much you eat

Cognitive Behaviour Therapy Class 2: Why do I eat the way I do?

- Building awareness on how thoughts and feelings influence dietary choices

Cognitive Behaviour Therapy Class 3: Self-monitoring and how to change your thinking

- How to be more aware of triggers for unhealthy dietary choices and how to change your response

Cognitive Behaviour Therapy Class 4: Self-esteem

- How to get back on track after a slipping back into old habits

Cognitive Behaviour Therapy Class 5: Boosting the belief that you can do it

- How to create an environment to support ongoing success

Cognitive Behaviour Therapy Class 6: Incentives and mindful eating

- How to maintain positive change

Exercise intervention:

Home-based Exercise: In addition to the instructions on using Fitbit ®, at the start of the intervention, participants in the intervention group received a 30-minute individualized, in-person consultation with an exercise health coach (certified kinesiologist) and received a personalized home exercise program for months one to four of the intervention. The four-month home exercise program started with a minimum of three sessions per week of 20 minutes of physical activity, progressing to five sessions per week of 40 minutes of physical activity (a total of 200 mins/week). The consultation also taught participants how to monitor their heart rate and keep within their individualized target heart rate zone using the Fitbit ®. Each participant was given an exercise log sheet to record the type, intensity, and duration of exercise along with their peak heart rate each week. At the weekly nutrition classes, participants handed in their exercise log sheets to encourage adherence. If there were any issues with their Fitbit ®, these were addressed in the nutrition classes.

Supervised Exercise and Education: Cardiac rehabilitation (CR) took place twice-weekly during months four to six and included moderate-intensity interval training at the Richmond Hospital Healthy Heart program. Before starting in-center exercise, each participant received an individualized 30-minute consult with the CR nurse and CR physiotherapist who ran the program and who were present at all classes to determine individual goals and limitations. In addition, volunteer university students helped participants track their exercise intensity and duration and ensured a 2:1 ratio of participants to staff. Polar chest strap heart rate monitors (Polar Electro, Kempele, Finland) were used for all participants during in-house exercise. Each cohort of 20 was divided into groups of 10 for exercise sessions. The curriculum included three nutrition classes (in addition to those described above), four stress management classes, and one pharmacy education class.

Exercise protocol: One-hour education class occurred weekly after the CR exercise session. At check-in the nurse measured resting BP and HR. If there was any concern of arrhythmia an ECG strip was done. Patients were allowed to exercise in atrial fibrillation as long as

exercise HR were kept below 85% of their HRpeak achieved during their graded exercise test (at baseline).

Participants performed 10 minutes of a group warm up activity. Following the warm up, participants performed supervised interval training on their preferred equipment (treadmill, stationary bike, recumbent bikes, recumbent steppers, elliptical machines, and rowing machines). University students were present to monitor and record HR, exercise level and time so that the ratio of participant to staff was at maximum 2 participants to 1 staff. Before beginning the intervals, participants exercised for 5 min at 65% of HRpeak. The interval protocol consisted of 5 sets of the following: 2 min at high intensity (65-85% HRpeak), then 2 min lower intensity (60-65% HRpeak) intervals for a total 20 min. With each set participants would be encouraged to increase their intensity as tolerated using HR as a guide. The goal was to obtain 85% HRpeak. If any concerns of arrhythmia arose, the nurse recorded BP and ECG rhythm strip and the cardiologist was consulted when necessary. After individual exercise there was a 5 min group cool down. Following the interval session, participants performed 10 minutes of resistance training. Resistance training was individualized to take into account participant restrictions. Initially, participants performed low-intensity, isometric exercises (using own body weight) focusing on core and major muscle groups, and gradually the intensity of the exercises increased over the course of the exercise intervention. The exercise session finished with a cool-down period (at self-selected intensity). Total exercise session time is just under 60 minutes.

<table>
<tr><th>Week #</th><th>Nutrition Education and Food Skills</th><th>Cognitive Behaviour Therapy</th><th>Home-based Exercise Program</th><th>Home-based Walking Program</th><th>Cardiac Rehabilitation (Supervised Exercise)</th></tr>
<tr><td>1</td><td>1hr consult + 1hr/week</td><td rowspan="4">--</td><td>1hr consult + 20min x 3/week</td><td rowspan="7">≥30min x 5/week</td><td rowspan="7">--</td></tr>
<tr><td>2</td><td rowspan="3">1hr/week</td><td>20min x 3/week</td></tr>
<tr><td>3-4</td><td>25min x 3/week</td></tr>
<tr><td>5-6</td><td>30min x 3/week</td></tr>
<tr><td>7-8</td><td rowspan="3">--</td><td rowspan="3">1hr/week</td><td>30min x 4/week</td></tr>
<tr><td>9-11</td><td>40min x 4/week</td></tr>
<tr><td>12-16</td><td>50min x 4/week</td></tr>
</table>

17	1hr consult	--	--		2hr/week
18-24	--				
25-52				≥30min x 5/week<br>*Optional weekly walking group	--

Comorbidity referral and targets.

1. Diabetes: Based on *Diabetes Canada 2018 Clinical Practice Guidelines for the Prevention and Management of Diabetes in Canada*)[19]
    a. Refer if A1c >6.5 % or requested by cardiologist
    b. Initial A1c 6.5-8%
        i. Start Lifestyle
        ii. Metformin if A1c >6.5% at 3mo
    c. Initial A1c >8%
        i. Start Metformin + Lifestyle
        ii. A1c not controlled at 3 month: Add DPP4
    d. Metabolic decompensation or symptomatic hyperglycemia
        i. Start insulin
        ii. Home Glucose monitoring
    e. See Patients q3mo, for titration, unless other concerns
2. Hypertension: Based on Hypertension Canada’s 2018 guidelines for diagnosis, risk assessment, prevention, and treatment of hypertension in adults and children [20]
    a. Refer if SPB >=130, DBP >=80 or requested by cardiologist
    b. If Framingham Risk >15%, Age >75, or clinical or sub-clinical CVD
        i. Initial Treatment >=130 target <120/80
    c. If Diabetes
        i. Initiate Treatment >=130, Target <130/80
    d. Others (including stroke, CKD)
        i. Initial BP>=140/90, target <140/90
    e. Therapy

i. Preferentially start ACE/ARB as initial therapy for all patients

ii. See patients Monthly until BP controlled

iii. Avoid use of non-dihydropyridine CCB and Beta Blockers

iv. Avoid use of Alpha blockers, hydralazine

3. Dyslipidemia: Based on 2016 Canadian Cardiovascular Society guidelines for the management of dyslipidemia for the prevention of cardiovascular disease in the adult[21]

   a. Refer if indications for therapy and patient is not on therapy or requested by cardiologist

      i. Established CAD/preclinical CAD

      ii. LDL >5

      iii. T2DM and Age >40 or disease x 15 years and age >30 or Microvascular disease

      iv. CKD (eGFR<60 or ACR >3) and Age >50

      v. AAA

      vi. Framingham >20%

      vii. Framingham 10-19% AND

         1. LDL >=3.5
         2. Non-HDL >=4.3
         3. ApoB>=1.2
         4. Men >=50/Women >=60 + additional CV risk factor

   b. Target

      i. LDL-C <2mmol/K or 50% decrease

      ii. ApoB <0.8g/L

      iii. Or non-HDL <2.6

   c. Treatment

      i. Rosuvastatin 10-40mg/day

      ii. uptitrate statin or add ezetimibe if not at target at initial visit

      iii. reassess lipids at 3 months: add statin or ezetimibe if not at target
